# The relationship between rehabilitation motivation and upper limb motor function in stroke patients

**DOI:** 10.3389/fneur.2024.1390811

**Published:** 2024-05-28

**Authors:** Wenxi Li, Guangyue Zhu, Yang Lu, Jinglei Wu, Zhuoxin Fu, Junyi Tang, Guohui Zhang, Dongsheng Xu

**Affiliations:** ^1^Department of Rehabilitation Medicine, Shanghai University of Traditional Chinese Medicine, Yueyang Hospital of Integrated Traditional Chinese Medicine and Western Medicine, Shanghai, China; ^2^Department of Rehabilitation, Shanghai University of Traditional Chinese Medicine, Shanghai, China; ^3^Engineering Research Center of Traditional Chinese Medicine Intelligent Rehabilitation, Ministry of Education, Shanghai, China

**Keywords:** rehabilitation, stroke, motivation, upper limb motor function, activities of daily life (ADL)

## Abstract

**Objective:**

Insufficient motivation among post-stroke survivors may be an important factor affecting their motor function recovery. This study seeks to investigate the relationship between motivation and functional recovery in stroke patients undergoing rehabilitation training.

**Materials and methods:**

103 stroke patients with upper limb impairments were studied during their hospital stays. Assessments were done before and after rehabilitation training to measure motivation, emotional state, motor function, and independence in daily activities. Data analysis was conducted to examine the distribution of these factors among the participants. Pearson and Spearman correlation analyses were used to study the relationships between motivation, emotional state, and motor function. Patients were divided into high and low motivation groups based on the Rehabilitation Motivation Scale (RMS), and chi-square and rank-sum tests were used to compare functional differences before and after treatment among patients with varying levels of motivation.

**Results:**

66 participants were found to have low motivation in the initial assessment of the RMS (64.08%). Consistency in motivation levels was observed among patients with high motivation (*r* = 0.648, *P*<0.001). Apathy was identified as the main factor affecting motivation in patients with low motivation (*p* = 0.027), while depression and anxiety were not significantly correlated. Motivation was strongly linked to improvements in upper limb motor function, daily living activities, and self-exercise duration (*p* < 0.001) for stroke patients undergoing rehabilitation. Post-training, there was a notable increase in motivation, motor function, and independence in daily activities (*p* < 0.001). Increased rehabilitation motivation was linked to better upper limb motor function and daily independence in patients, particularly those with low motivation. This correlation was significant for both the FMA-UE and FIM scores.

**Discussion:**

Old patients with poor upper limb motor function often have low motivation, which hinders their recovery. Using strategies to boost motivation in stroke patients with impaired upper limb function could greatly improve their rehabilitation and motor skills. It is crucial to prioritize these intervention strategies.

**Conclusion:**

Enhancing rehabilitation motivation in stroke patients with low motivation and upper limb motor impairments can foster the restoration of their functional capabilities.

## 1 Introduction

Stroke is characterized by high incidence, high mortality, and high disability rates (1). In China, its prevalence has been increasing annually, currently ranking first among the top 10 deadly and disabling diseases (2). After a stroke, 70% of patients experience varying degrees of functional impairment and require rehabilitation treatments for gradual recovery (3). Nearly half of the survivors suffer from impaired upper limb function, severely affecting their daily lives. The recovery period for stroke-induced upper limb function is lengthy, and intensive, repetitive training is key to functional improvement (4). However, the intensity and duration of rehabilitation training for most stroke patients have not reached the levels necessary for significant functional improvement (5, 6), one crucial reason being the lack of sufficient rehabilitation motivation. Patients’ lack of initiative in engaging in unsupervised rehabilitation training may be related to decreased motivation, a situation that becomes more pronounced 6 months after stroke onset (7). Motivation is defined as an orientation that generates and sustains behavior in humans and other animals to achieve a certain goal (8). In the context of rehabilitation training, motivation primarily manifests as the patient’s initiative to participate in rehabilitation tasks, the extent of their involvement during rehabilitation, and their willingness to dedicate time to rehabilitation training (9). Rehabilitation motivation is influenced by a variety of factors, including internal and external environmental aspects, with contributing factors to its decline possibly related to individual characteristics (such as cognition and emotion) and external support factors (10).

Current research indicates that low rehabilitation motivation can lead to patients refusing or reducing their participation in rehabilitation training, resulting in decreased exercise time and frequency (11). This can cause sedentary lifestyle and limit the level of participation in daily activities (12). However, there is a lack of literature supporting whether low motivation affects the recovery of upper limb function. To enhance the participation level of stroke patients in rehabilitation, strategies such as game approaches, rehabilitation robots, and virtual reality (VR) equipment have been incorporated into rehabilitation training (13–17). These methods aim to increase enjoyment and initiative, boost patient motivation, and thereby enhance the effectiveness of rehabilitation training. Nevertheless, these studies focus on the improvement of physical functions and do not specifically elaborate on changes in rehabilitation motivation. In addition to strategies that improve rehabilitation motivation through external factors, motivational interviewing, a technique that enhances rehabilitation motivation by mobilizing internal factors of patients, has also been widely used in clinical practice (18, 19). However, such research focuses only on changes in rehabilitation motivation, overlooking the impact that improvements in motor function may have on rehabilitation motivation.

Although strategies to enhance rehabilitation motivation have been applied in stroke rehabilitation, their primary goal is to promote functional recovery by improving motivation. However, current studies each focus on different aspects, typically demonstrating changes in either motivation or motor function independently, without establishing a direct link between the improvement in motivation and the recovery of upper limb motor function.

Therefore, this study aims to determine the correlation between rehabilitation motivation and upper limb motor function in stroke patients, providing a basis for the clinical application of strategies to enhance rehabilitation motivation in stroke rehabilitation.

## 2 Materials and methods

### 2.1 Study participants

Participants in this study were stroke patients hospitalized in the departments of Rehabilitation, Acupuncture, and Tuina at Shanghai University of Traditional Chinese Medicine Affiliated Yueyang Hospital of Integrated Traditional Chinese and Western Medicine. The included subjects met the diagnostic criteria for stroke (ischemic or hemorrhagic); were experiencing their first stroke episode; aged between 18 and 80 years; of any gender; right-handed; without severe cognitive and communication impairments (MMSE score ≥ 20). They agreed to participate and were willing to cooperate in a 30–45-min assessment process after fully understanding the study procedures. Key exclusion criteria included patients with severe systemic diseases that could not tolerate rehabilitation treatment; severe psychiatric conditions, major depression, anxiety; severe joint contractures; severe pain, sleep disorders, psychiatric disorders; and auditory or visual impairments that could affect assessment and treatment. This study was approved by the Ethics Committee of Shanghai University of Traditional Chinese Medicine Affiliated Yueyang Hospital of Integrated Traditional Chinese and Western Medicine (Ethical approval number: 2021–122, Clinical trial registration number: ChiCTR2300069068).

### 2.2 Experiment protocol and patient evaluation

#### 2.2.1 Experiment protocol

We conducted baseline assessments for 106 stroke patients eligible for rehabilitation treatment during their hospitalization and performed a second assessment 2 weeks after enrollment. 103 participants completed the full assessment and treatment process. At baseline, subjects were divided into low and high motivation groups based on their scores on the Rehabilitation Motivation Scale (RMS), and correlations with emotional and motor-related factors were analyzed according to motivation grouping. Furthermore, after 2 weeks of rehabilitation training, the impact of motivation on motor function recovery was explored based on the low and high motivation groupings.

Eligible subjects underwent rehabilitation training after inclusion, which consisted of conventional rehabilitation training and self-directed rehabilitation exercises. Conventional rehabilitation training included physical therapy and occupational therapy. Each patient was required to undergo 20 min of one-on-one physical therapy and 20 min of one-on-one occupational therapy 5 days a week, for a total of 2 weeks. The training under the one-on-one guidance of occupational therapists primarily aimed at guiding participants in targeted upper limb motor function training, such as grasping objects, combing hair, wiping tables, picking up peanuts, and buttoning. Self-directed rehabilitation exercises refer to the additional self-rehabilitation exercises that participants were encouraged to perform independently in the occupational therapy setting or their wards, in addition to the exercises under the therapist’s guidance. There was no limit on the exercise time, and the content could refer to tasks designed by the therapists. After completing 2 weeks of rehabilitation treatment, subjects were required to undergo a second assessment focusing on changes in rehabilitation motivation and motor function.

#### 2.2.2 Evaluation methods

During the baseline assessment, the study variables related to individuals included basic information, medical history, independent exercise time, and caregiving situation. We also used the Mini-mental State Examination (MMSE) as an indicator for cognitive function screening. In this study, subjects with a score of ≥20, indicating normal cognitive function or mild cognitive impairment and the ability to communicate normally, were included.

To ensure the accuracy of the assessment, we employed both self-assessment and observer-assessment tools to measure the subjects’ rehabilitation motivation. The Stroke Rehabilitation Motivation Scale (SRMS-7) is a self-assessment tool for stroke rehabilitation motivation, adapted from a movement motivation assessment tool (20). SRMS consists of 28 items exploring three domains (amotivation, extrinsic motivation, and intrinsic motivation). In this study, a shorter version of the SRMS with 7 items was used, which has been shown to have good reliability. The 7-item SRMS was developed by selecting the item with the best reliability from each subscale of the 28-item version, scored using a Likert 5-point scale. The total score range of the 7-item SRMS is 7–35, with higher scores indicating higher motivation. A score of ≥21 is considered normal to high motivation. The Rehabilitation Motivation Scale (RMS), developed by modifying and translating the RMS designed by Litman in 1961 into Chinese by Guo, is assessed by physical therapists through observation of patients’ behavior in participating in rehabilitation treatment to measure the strength of their rehabilitation motivation (21). It consists of 8 items scored using a Likert 4-point scale. The scale’s score range is 8–32, with scores ≥25 considered as normal or high motivation. In the second assessment, subjects were re-evaluated for motivation and motor function-related assessments to investigate the impact of rehabilitation training on motivation and movement in stroke patients and the correlation between motivation and motor function recovery.

The assessment of apathy levels was conducted using the Apathy Evaluation Scale, Clinician Version (AES-C), which evaluates an individual’s emotional experience and support level in participating in social support and partner relationships across dimensions of relationship, emotion, support, and participation. The total score range of the scale is 18–72, with scores above 35 typically considered indicative of apathy. The Hamilton Depression Scale (HAMD) and the Hamilton Anxiety Scale (HAMA) were used to assess the emotional states of the subjects. In the HAMD, scores of <8 are considered normal, 8–20 indicate possible depression, 21–35 suggest a likely diagnosis of depression, and > 35 indicate severe depression. This study excluded subjects with severe depression at the time of inclusion. In the HAMA, scores of less than 7 are considered normal, 7–14 may indicate anxiety, 14–21 confirm the presence of anxiety, 21–29 indicate marked anxiety, and scores greater than 29 indicate severe anxiety. Subjects with severe anxiety were excluded from this study. The Fugl-Meyer Assessment Upper Extremity Scale (FMA-UE) was used to evaluate the degree of motor function impairment in the hemiplegic upper limb of the subjects. The assessment of upper limb motor function includes 33 items, with a total score of 66. To assess the subjects’ independence in daily living, we used the Functional Independence Measure (FIM), which includes evaluations of self-care, mobility, control, communication, and social participation, among others, with a total score of 126. The lowest score is 18, indicating complete dependence; 19–35 points indicate very severe dependence; 36–53 points indicate severe dependence; 54–71 points indicate moderate dependence; 72–89 points indicate mild dependence; 90–107 points indicate conditional independence; 108–125 points indicate near independence; and 126 points indicate complete independence.

### 2.3 Statistical analysis

The results of this study were statistically analyzed using Origin Pro 2021 (Chinese version v9.8.5.204) software. Data collection, organization, and statistical analysis were performed by independent researchers. The RMS was assessed by occupational therapists who administered treatment to the subjects, while other assessments were conducted by independent evaluators. The primary analysis focused on the results of subjects who completed the baseline assessment, received rehabilitation treatment, and also completed the second assessment. In the baseline characteristics of the subjects, categorical information characteristics (gender, affected side, underlying diseases, diagnosis, caregiving situation) were represented as ratios. After dividing subjects into low and high motivation groups based on the RMS, the Chi-squared test was used to compare the proportion distribution of these aspects. Baseline characteristics such as age, disease duration, independent exercise time, MMSE, HAMA, and HAMD were represented as medians (interquartile range), and data were compared between low and high motivation groups using the Mann–Whitney test. The Shapiro–Wilk test was used to assess the normality of the data. For continuous data that were not normally distributed (including AES-C, RMS, SRMS-7, FMA-UE, FIM), the Wilcoxon test was used for before-and-after comparisons. Pearson correlation analysis and Spearman correlation analysis were used to elucidate the correlations between motivation and emotional and motor functions. A *p* value of <0.05 was considered statistically significant.

## 3 Results

### 3.1 Demographic and baseline clinical characteristics

This study initially included 106 participants, with 3 dropouts (2 due to withdrawal of consent by the patients, and 1 lost to follow-up), resulting in 103 participants completing all assessments. The baseline characteristics are presented in Table 1. The participants included in this study did not exhibit significant cognitive impairments. None of the participants had depression or anxiety. Only a minority of participants reported being able to live independently during their hospital stay, with the rest requiring care from family members or caregivers. Participants were grouped according to their scores on the RMS, and there were statistically significant differences between the two groups in terms of gender, baseline characteristics of HAMA and HAMD (*P*<0.05), as well as in the differences in independent exercise time and MMSE scores (*P*<0.001).

**Table 1:** Baseline demographic and clinical characteristics.

Characteristics	Total (*n* = 103)	Motivation(*n* = 103)		Statistics	*p* value
		Lower motivation(*n* = 66)	Higher motivation(*n* = 37)		
Age (years)	66.00 (39.00, 71.00)	66.00 (58.00, 71.00)	68 (61.00, 71.00)	−0.114	0.909
Gender (male/female)	72/31	43/23	29/8	8.440	0.004^**^
Affected side (left/right)	69/34	49/17	20/17	0.033	0.855
Disease duration (months)	15.00 (5.00, 36.00)	21.00 (6.00, 41.25)	12.00 (4.00, 40.00)	−0.898	0.370
Hypertension (yes/no)	86/17	53/13	33/4	1.046	0.306
Diabetes (yes/no)	42/61	27/39	15/22	0.794	0.373
Independent exercise time (hours)	0.50 (0.50, 1.00)	0.50 (0.00, 1.00)	1.00 (1.00, 2.00)	4.767	*p* < 0.001^***^
Diagnosis (cerebral infarction/cerebral hemorrhage)	88/15	57/9	31/6	1.671	0.196
Care situation (independent/family or one-on-one care/one to several care)	27/48/28	16/32/18	11/16/10	1.087	0.896
MMSE	27.00 (26.00, 29.00)	27.00 (25.00, 28.00)	29.00 (26.00, 30.00)	−3.269	*p* < 0.001^***^
HAMD	3.00 (1.00, 6.00)	3.00 (2.00, 6.00)	2.00 (0.00, 5.00)	2.080	0.037^*^
HAMA	4.00 (1.00, 7.00)	5.00(2.00, 9.00)	2.00 (1.00, 5.00)	2.531	0.011^*^
AES-C	33.00 (26.00, 32.00)	37.00 (30.00, 42.50)	26.00 (24.00, 32.00)	5.581	*p* < 0.001^***^
FMA-UE	35.00 (14.00, 56.00)	21.50 (11.75, 48.50)	52.00 (29.00, 60.00)	−3.517	*p* < 0.001^***^
FIM	116.00 (100.00, 121.00)	110.50 (89.00, 121.00)	119.00 (115.00, 123.00)	−3.461	*p* < 0.001^***^

^*^p < 0.05, ^**^p < 0.01, ^***^p < 0.001.

**Figure 1:**
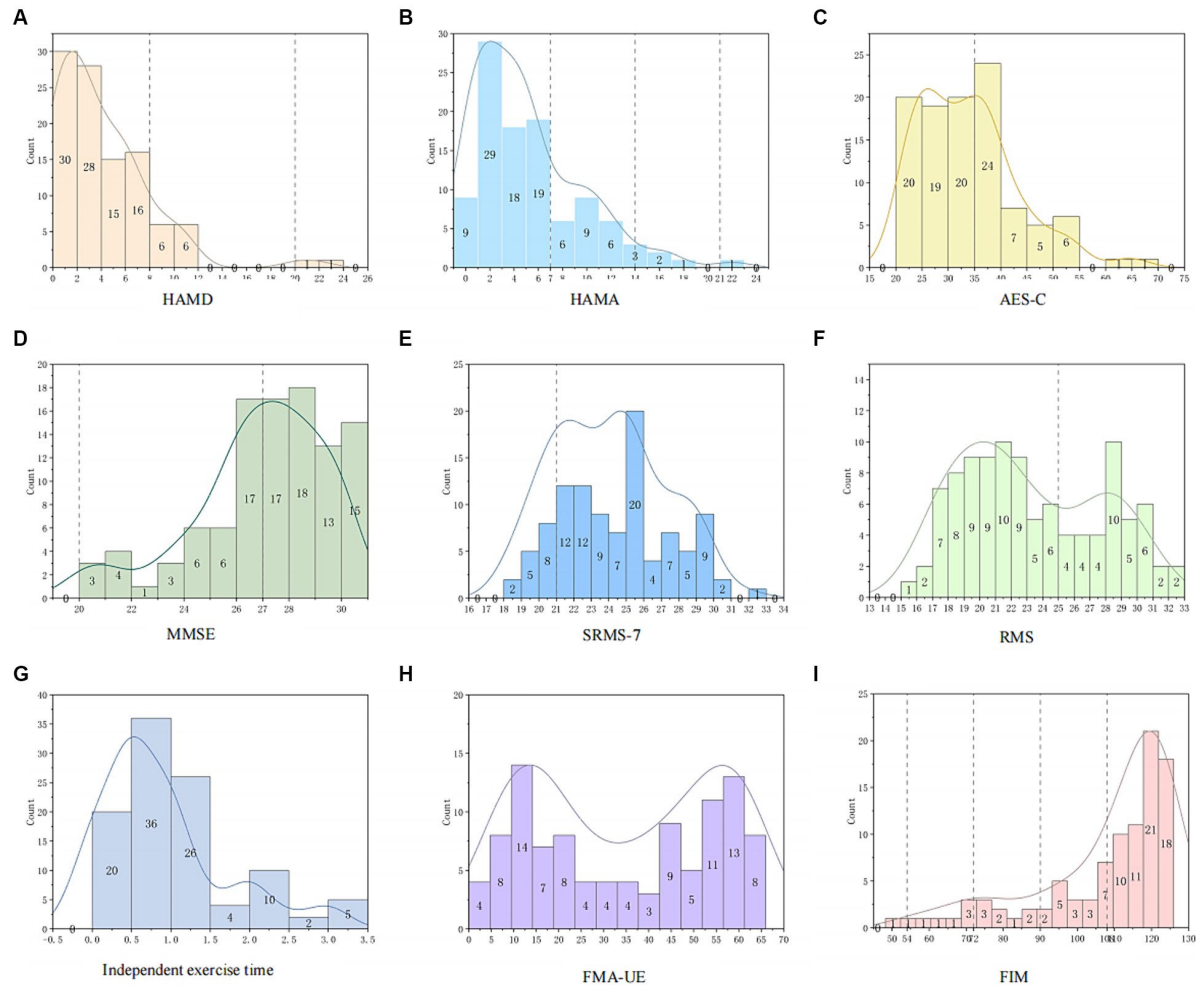
Distribution characteristics of emotion, motivation, and motor function. **(A)** The dashed lines represent 8 and 20 points, respectively. **(B)** The dashed lines represent 7, 14, and 21 points. **(C)** The dashed line represents 35 points. **(D)** The dashed line represents 27 points. **(E)** The dashed line represents 21 points. **(F)** The dashed line represents 25 points. **(G)** The distribution characteristics of self-exercise time for all subjects. **(H)** The distribution characteristics of FMA-UE for all subjects. **(I)** The dashed lines represent 54, 72, 90, and 108 points.

Figure 1 illustrates the distribution characteristics of variables related to emotion, motivation, and motor function at baseline. The curve fitting method used in the figures is kernel smooth. In terms of cognitive function (Figure 1D), MMSE assessment showed that 38.83% (22) of participants had mild cognitive impairments, while the rest were normal. Regarding emotional aspects, only 0.97% (14) of patients were likely to have depression (Figure 1A), and 27.18% (23) of patients were likely to have anxiety (Figure 1B), with no participants exhibiting severe depression (>35 points) or severe anxiety (>29 points). According to the AES-C, 42.72% (24) of participants had scores ≥35, indicating possible clinical signs of apathy (Figure 1C). Regarding rehabilitation motivation, 14.56% of individuals (15) considered their rehabilitation motivation to be low according to the SRMS-7 (Figure 1E). In the observer-assessment RMS (Figure 1F), 64.08% (66) of participants were considered by therapists to have scores below 25 at the initial assessment, indicating a decline in rehabilitation motivation. In terms of motor function, FIM results indicated that approximately 58.25% (60) of participants could achieve basic independence, 19.42% (20) were conditionally independent, 7.77% (8) mildly dependent, 6.80% (7) moderately dependent, and 1 person was severely dependent (Figure 1I). FMA-UE scores (Figure 1H) showed that baseline scores for upper limb motor function were mainly concentrated in two ranges: 5–25 points and 45–66 points. Regarding the time spent on independent exercise (Figure 1G), most patients exercised for 0–1.5 h.

### 3.2 Assessment of motivation

Rehabilitation motivation is currently assessed primarily through scales, which can be divided into self-assessment and observer-assessment types. There may be significant differences between individual scores on self-assessment and observer-assessment motivation scales. Therefore, we compared the correlation between self-assessment and observer-assessment scales to examine the accuracy of scale assessments in evaluating motivation. Results showed good consistency between SRMS-7 and RMS.

**Figure 2:**
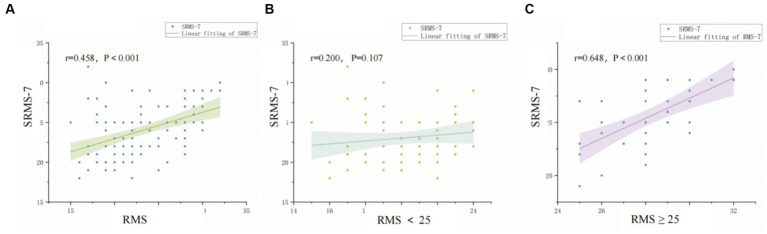
Correlation between self-assessed and observer-assessed motivation. **(A)** Correlation between RMS and SRMS-7 in total patients. **(B)** Correlation between RMS and SRMS-7 in the low motivation group. **(C)** Correlation between RMS and SRMS-7 in the high motivation group.

As shown in Figure 2, Figure 2A displays the relationship between SRMS-7 and RMS through scatter plots. Using Spearman correlation to determine the relationship between SRMS-7 and RMS, we found *r* = 0.464, *P*<0.001, indicating a positive correlation between the two. Figure 2B shows the scatter plot of the correlation between SRMS-7 and RMS for participants considered to have low rehabilitation motivation (scores below 25 on RMS). Using Spearman correlation analysis, *r* = 0.200, *p* = 0.107, indicating no correlation between the two. Figure 2C analyzes the correlation between RMS scores (≥25 points) and SRMS-7. Spearman correlation analysis resulted in *r* = 0.648, *P*<0.001, indicating a positive correlation between the two.

### 3.3 Correlation between motivation, emotion, and motor function

**Figure 3:**
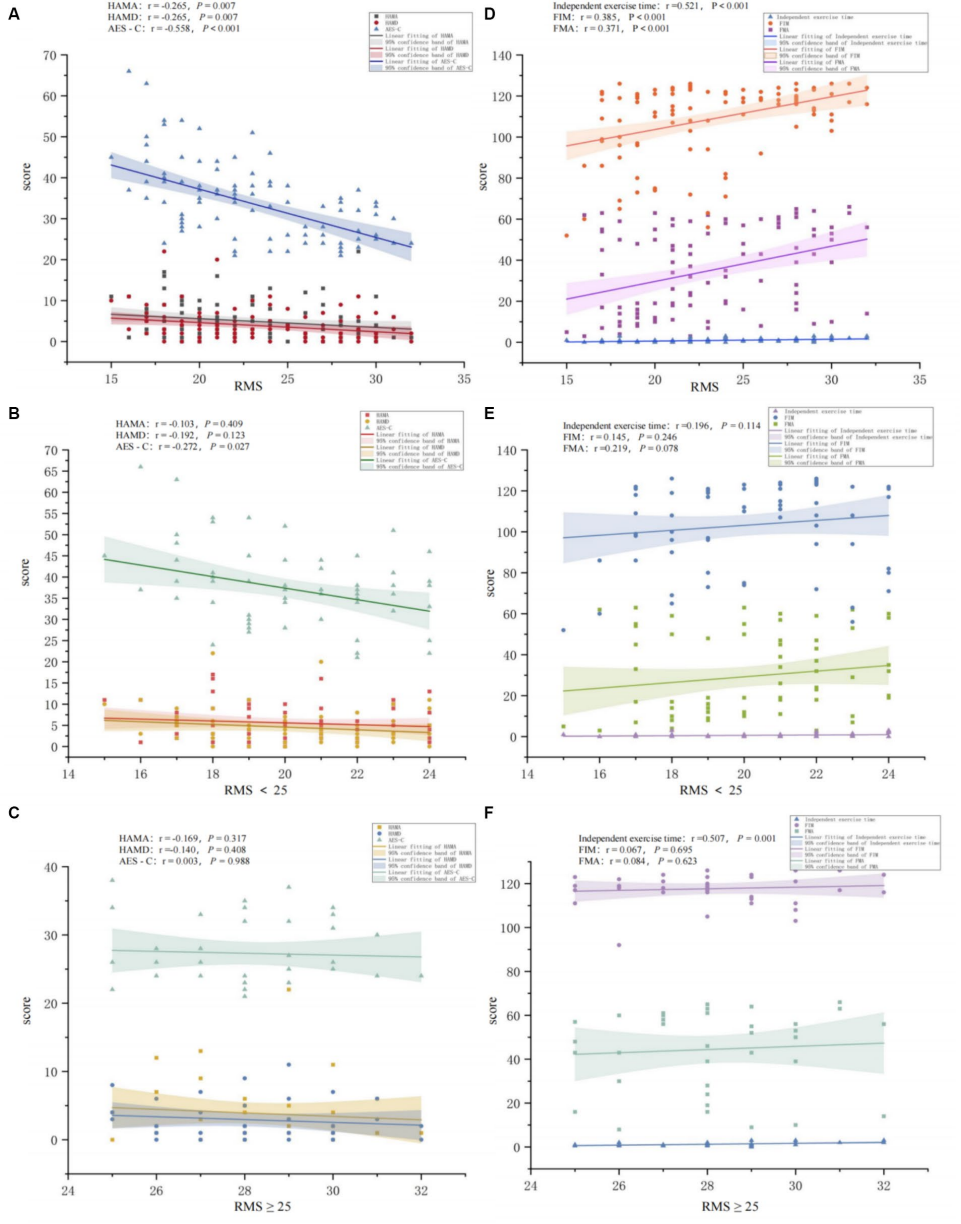
Correlation between RMS and emotion (ACE-S, HAMA, HAMD) and motor function (FMA, FIM, independent exercise time). **(A)** The figure shows the correlation between RMS and emotion among all study participants. **(B)** The figure shows the correlation between RMS and emotion among participants in the low motivation group. **(C)** The figure shows the correlation between RMS and emotion among participants in the high motivation group. **(D)** The figure shows the correlation between RMS and motor function among all study participants. **(E)** The figure shows the correlation between RMS and motor function among participants in the low motivation group. **(F)** The figure shows the correlation between RMS and motor function among participants in the high motivation group.

In this study, we analyzed the correlation between motivation and emotion (HAMA, HAMD, AES-C) and motor function (FMA-UE, FIM, independent exercise time) to examine their relationships. The results showed that motivation was negatively correlated with apathy, anxiety, and depression; and positively correlated with motor function and the ability to perform activities of daily living. Figure 3 uses scatter plots to depict the distribution of correlations between motivation, emotional state, and motor function, further elucidated by Spearman correlation coefficients. Figure 3A shows the relationship between motivation and emotions (HAMA, HAMD, AES-C) among all participants, revealing negative correlations between motivation and depression, anxiety, and apathy, with statistically significant correlations (HAMD *p* = 0.007, HAMA *p* = 0.007, AES-C *P* <0.001). Additionally, motivation was positively correlated with upper limb motor function (FMA-UE), the ability of daily livings (FIM), and independent exercise time among all participants, with statistically significant correlations (*P* <0.001). In the low motivation group (Figures 3B,E), motivation was negatively correlated with apathy, with statistical significance (*p* = 0.027), while no significant correlation was found with depression and anxiety. There was no significant correlation between low motivation and motor function. In the high motivation group (Figures 3C,F), no significant correlations were found between motivation and emotional factors. When analyzing the correlation with motor function, motivation was not significantly correlated with FMA-UE and FIM, but was positively correlated with independent exercise time, showing significant correlation (*P* <0.05).

### 3.4 The impact of rehabilitation training on motivation and motor function

**Figure 4:**
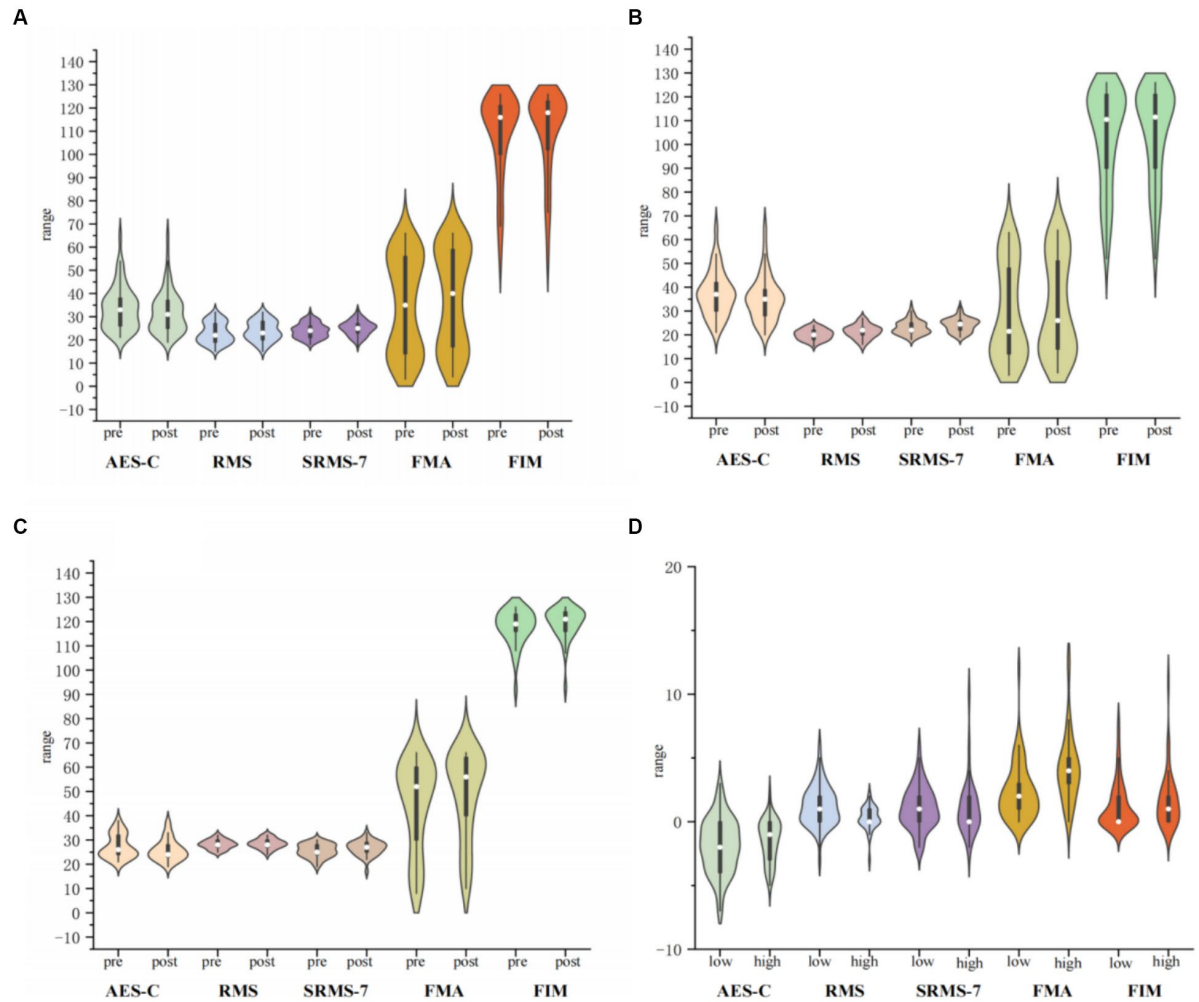
Impact of rehabilitation training on emotion (ACE-S), motivation (RMS, SRMS-7), and Motor Function (FMA, FIM). **(A)** The figure shows the changes before and after rehabilitation training for all study participants. **(B)** The figure shows the changes before and after rehabilitation training for participants with low motivation. **(C)** The figure shows the changes before and after rehabilitation training for participants with high motivation. **(D)** The figure shows the differences in changes after rehabilitation training between the low and high motivation groups.

Figure 4 displays the changes in apathy, rehabilitation motivation, upper limb motor function, and independence in daily activities among study participants after undergoing rehabilitation training. All participants showed significant improvements in apathy, rehabilitation motivation, upper limb motor function, and independence in daily activities after 2 weeks of rehabilitation training (*P* <0.001) (Figure 4A). This significant improvement trend was consistent among both the low and high motivation groups (Figures 4B,C). When comparing the extent of improvement in AES-C, RMS, FMA-UE, and FIM between the low and high motivation groups, it was found that there were no significant differences in improvements in AES-C, SRMS-7 and FIM. However, significant differences were observed in the improvements in RMS (*P* <0.05) and FMA-UE (*P* <0.001).

### 3.5 The impact of motivation on the improvement of patients’ motor function

**Figure 5:**
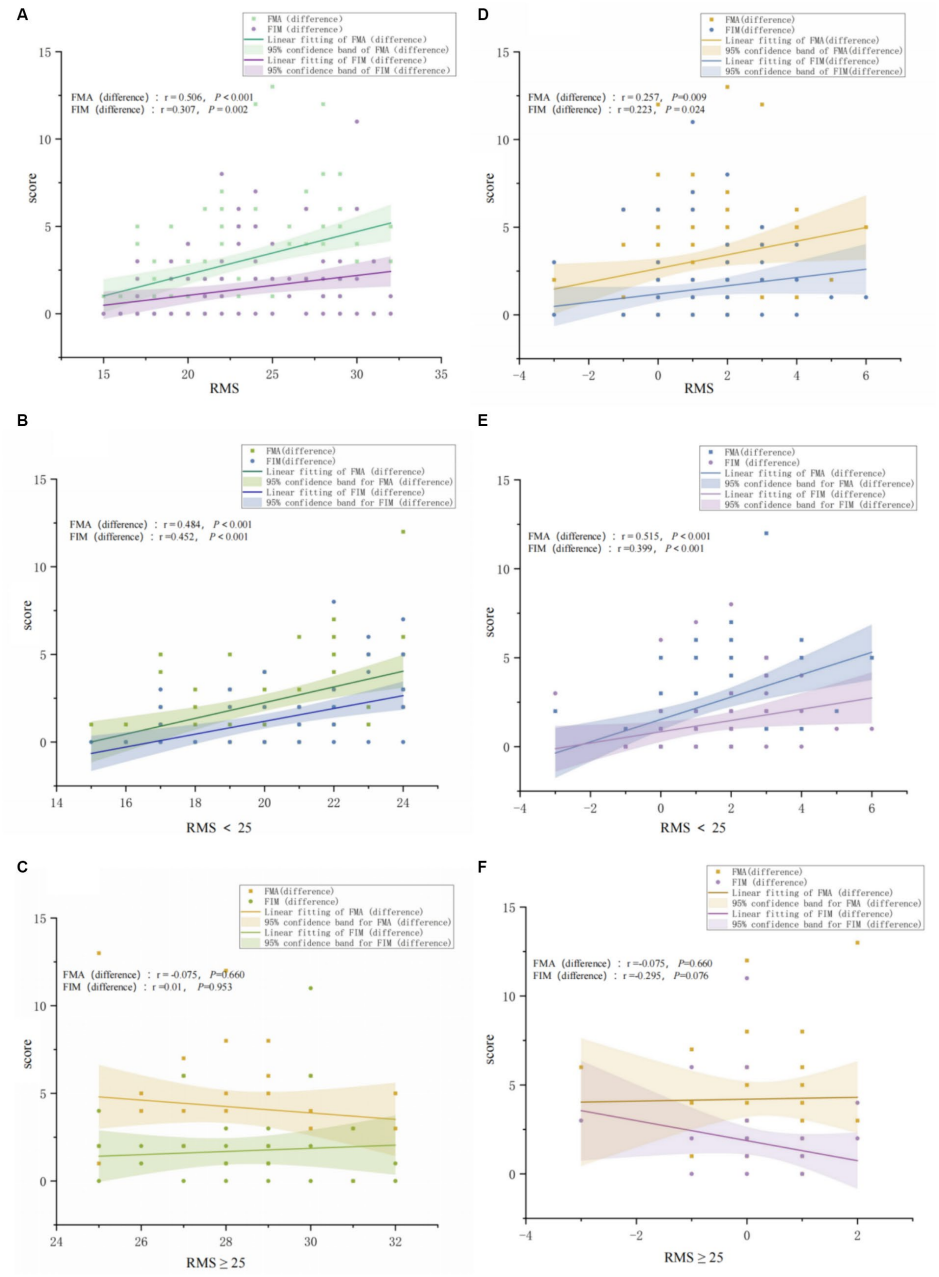
The impact of high vs. low Motivation on the improvement of patients’ upper limb motor function (FMA-UE) and independence in daily living (FIM). **(A)** The figure shows the correlation between the initial RMS results and FMA-UE and FIM among all study participants. **(B)** The figure shows the correlation between the initial RMS results and FMA-UE and FIM among participants with low motivation. **(C)** The figure shows the correlation between the initial RMS results and FMA-UE and FIM among participants with high motivation. **(D)** The figure shows the correlation between the change in RMS before and after rehabilitation training and FMA-UE and FIM among all study participants. **(E)** The figure shows the correlation between RMS before and after rehabilitation training and FMA-UE and FIM among participants with low motivation. **(F)** The figure shows the correlation between the change in RMS before and after rehabilitation training and FMA-UE and FIM among participants with high motivation.

Figure 5 displays the correlation between patients’ rehabilitation motivation and the degree of improvement in FIM and FMA-UE, indicating that an increase in motivation among patients with low motivation may facilitate improvements in motor function. Overall (Figures 5A,D), the level of rehabilitation motivation was positively correlated with improvements in upper limb motor function and enhancements in independence in daily activities (FMA-UE (difference) *r* = 0.506, FIM (difference) *r* = 0.307), with significant correlations (FMA-UE (difference): *P*<0.001; FIM (difference) *p* = 0.002). Improvements in rehabilitation motivation were also positively correlated with enhancements in upper limb motor function and independence in daily activities (FMA-UE (difference) *r* = 0.257, FIM (difference) *r* = 0.233), showing significant correlations (FMA-UE (difference): *p* = 0.009; FIM (difference) *p* = 0.024). In the low motivation group (Figures 5B,E), the initial assessment of rehabilitation motivation was significantly related to improvements in upper limb function and enhancements in independence in daily activities (FMA-UE (difference): *P*<0.001; FIM (difference) *P*<0.001). Furthermore, in the low motivation group, the increase in rehabilitation motivation after undergoing rehabilitation training was significantly positively correlated with improvements in FMA-UE and FIM (FMA-UE (difference): *r* = 0.515, *P*<0.001; FIM (difference): *r* = 0.399, *P*<0.001), indicating that the greater the increase in motivation, the higher the degree of motor function improvement. However, in the high motivation group (Figures 5C,F), the correlation between motivation assessment and improvements in upper limb motor function and independence in daily activities was not significant, and there was no significant correlation between improvements in motivation and functional improvements after rehabilitation training.

## 4 Discussion

Motivation plays a crucial role in the rehabilitation of stroke patients with motor function impairments, yet the distribution of motivation among stroke patients and its specific relationship with motor recovery remain unclear. By assessing and analyzing the relationships between motivation, emotion, and motor function in stroke patients, this study found that there is a general lack of rehabilitation motivation among patients with stroke-induced motor function impairments. The analysis of the correlation between motivation, emotion, and motor function revealed no significant correlation between motivation and anxiety or depression, but a significant correlation with the apathetic state of patients; moreover, patients with lower motivation also had lower initial motor function states, yet the improvements in motor function brought about by rehabilitation training positively correlated with motivation.

### 4.1 Distribution of motivation and assessment of motivation

The level of motivation is greatly related to subjective perception. To differentiate the motivation levels among participants, we employed both self-assessment and observer-assessment tools. The results revealed that only a small portion of participants (14.56%) considered their rehabilitation motivation to be low. However, nearly two-thirds of participants were deemed to have low motivation according to the observer-assessment motivation scale, suggesting potential differences between self-assessed and observer-assessed rehabilitation motivation tools. This discrepancy might be due to patients evaluating their motivation based on personal feelings (25, 26), whereas therapists assess patients’ motivation based on their engagement in activities, attitude towards rehabilitation, and compliance (27–29). In the correlation analysis between self-assessed and observer-assessed motivation scales, we found good consistency in assessments among participants with high motivation, but no correlation in assessments among participants with low motivation. This may be because patients with low motivation cannot accurately describe their state. Previous studies mostly used a single scale (self-assessment scale) to evaluate patients’ motivation (10, 18, 23, 30), which might miss some stroke populations with low motivation, hindering screening and timely intervention. As shown by the results of this study, combining self-assessment and observer-assessment scales is beneficial for identifying patients with low motivation. Additionally, scale assessments have limitations, and more objective tools for assessing rehabilitation motivation in stroke recovery are yet to be explored.

### 4.2 The relationship between emotion and motivation

It is commonly believed that low rehabilitation motivation following a stroke may be significantly influenced by emotional factors such as depression and anxiety. However, in this study, our analysis of the correlation between motivation and depression and anxiety found that rehabilitation motivation is not significantly related to depression and anxiety, but rather apathy is the most significant influencing factor on motivation. In the research conducted by Green et al. (31), although depression and apathy share common features such as a loss of pleasure, and patients with depression may also exhibit clinical signs of apathy, the main clinical characteristic of depression is accompanied by significant negative emotions, while apathy does not display significant negative emotional expressions. Its main features are a lack of drive, goal-directed behavior deficits, which are closely related to reduced motivation (32), consistent with our research findings. In this study, participants with low motivation showed significantly different performances on the apathy assessment scale compared to those with normal motivation, and participants with low motivation generally exhibited signs of apathy, whereas this was not observed in participants with high motivation. The theoretical basis for post-stroke apathy is the impairment of the brain network for goal-directed behavior (GDB) (32), with core structures being the anterior cingulate cortex (ACC) and nucleus accumbens (NAc) (33), which are closely related to decision-making regarding rewards, attention control, and reinforcement learning (34). The GDB-related brain network is regulated by dopaminergic neurons, and disruption or damage to this network makes it difficult for reward-related signals to be integrated and transmitted in structures such as the ACC, ventral striatum (*VS*), and prefrontal cortex (PFC) (35), leading to a reduction in approach behavior following perception of rewards, thus manifesting as reduced GDB and emotional apathy (36, 37). Additionally, there is a certain correlation between low motivation and cognitive function levels (38), although participants included in the study did not have significant cognitive impairments, those in the low motivation group had lower cognitive levels than those in the high motivation group. Patients with post-stroke apathy who further develop may also experience cognitive impairments (39), which is very unfavorable for the functional prognosis of stroke patients.

### 4.3 The relationship between upper limb motor function and motivation

In terms of motor function, we found that the level of rehabilitation motivation significantly affects the time spent on independent exercise. Participants with high motivation are willing to spend more time on self-exercise, with most dedicating at least 1 h per day to independent exercise. The higher the motivation, the longer the potential time spent on independent exercise, whereas participants with low motivation generally exercise for about 0.5 h. Wissink et al. (4) also mentioned that stroke patients with higher motivation during hospitalization participate in rehabilitation treatments with higher intensity, which is more conducive to successful discharge, aligning with our findings. At the time of enrollment, there were significant differences in FMA-UE and FIM between participants with low and high motivation. Compared to participants with high motivation, those with low motivation had poorer upper limb function and lower independence in daily living. This suggests that the level of motor function may have a certain impact on rehabilitation motivation; patients with poorer upper limb function participate less in daily activities and are more likely to exhibit low motivation. Previous studies have mainly explored the correlation between rehabilitation motivation and independence in daily living (40, 41). However, upper limb motor function has a crucial impact on the participation of stroke survivors in daily living activities. The level of upper limb motor function can lead to different expectations for independent living, potentially affecting the internal factors of patients’ rehabilitation motivation. This potential correlation has not received much attention.

On the other hand, besides focusing on the impact of stroke patients’ motivation on their participation duration in rehabilitation treatment, this study further explored the correlation between motivation and the effectiveness of upper limb rehabilitation. We found that participants showed significant improvements in both motivation and motor function after undergoing 2 weeks of rehabilitation training. This may suggest that rehabilitation training not only promotes improvements in motor function but also has a positive effect on enhancing rehabilitation motivation. Rehabilitation training increases the participation level of stroke survivors through individualized goal-directed task design (22), while simultaneously integrating game strategies with wearable devices (such as exoskeleton robots, gloves, VR glasses, etc.) (16, 42–44), enhancing patient engagement in therapy during upper limb training. Participants in the low motivation group showed greater improvements in both motivation and upper limb motor function (FMA-UE) after rehabilitation training than those in the high motivation group. This could be because participants in the high motivation group already scored higher in motivation and motor function compared to those in the low motivation group, leading to a smaller margin for improvement due to a potential ceiling effect. However, there is potential for improvement in motivation and motor function among the low motivation group, which was promoted after rehabilitation intervention. In the low motivation group, improvements in rehabilitation motivation were significantly positively correlated with enhancements in upper limb motor function and independence in daily living. This implies that, in addition to conventional strategies for improving motor function, applying strategies to enhance rehabilitation motivation during rehabilitation could also promote improvements in upper limb motor function for the low motivation group, potentially providing a boost. However, this approach may not be as meaningful for the high motivation group.

From a functional perspective, motor control requires the analysis and understanding of the movement before its execution, followed by the planning and implementation of the movement. In the advanced cognitive stage of motor control, motivation can encourage patients to focus more actively on understanding the movement and quickly translate it into practical actions (24). From a structural perspective, motor control and cognition share a common neural circuit basis. Regions such as the dorsolateral prefrontal cortex (dlPFC), dorsomedial prefrontal cortex (dmPFC), premotor cortex(PMC), and supplementary motor area(SMA) are key brain regions for motor control. The coordination of these brain regions ensures the smooth flow of motor processes (45). The neural circuits involved in cognition, especially motivation, are mainly concentrated in the PFC, sharing common key brain regions with motor control. Basic research results have also confirmed these views. Structure is the basis of function, and the reward circuit and motor system should share a common structural basis, starting from the core structures of the reward circuit, the NAc, and VTA. It has long been believed that the NAc does not directly participate in motor control until Sawada et al. corrected this view in their research. Their study on fine motor control after spinal cord injury in macaques, confirmed by intracranial electrode recordings, demonstrated a direct correlation between the electrical activities of the NAc and the motor cortex. In the early stages of injury, activating or inactivating the NAc can directly affect fine motor control (46). Therefore, this further illustrates the regulatory role of motivation in motor control.

### 4.4 Strengths and limitations

This study is the first to investigate the distribution and correlation between rehabilitation motivation, emotions, and motor function in stroke patients. The findings confirm the close correlation between motor function and rehabilitation motivation in stroke patients, providing clinical evidence for the application of motivation enhancement strategies in stroke recovery. In the future, we will further optimize and promote the application of an integrated rehabilitation program.

One of the main limitations of this study is that the research subjects should include more participants from different regions. In the next stage of the study, we will conduct multi-center clinical research, while further exploring the underlying mechanisms of the influence of motivation on motor function in stroke patients.

## 5 Conclusion

There is a correlation between rehabilitation motivation and upper limb motor function in stroke patients. Those with diminished motor function in their upper limbs tend to display a lower level of motivation, which negatively impacts the recuperation of their upper limb abilities. Beyond conventional rehabilitation methods, the adoption of strategies aimed at boosting rehabilitation motivation in stroke patients characterized by low motivation and impaired upper limb function could lead to improvements in both their desire for rehabilitation and their motor capabilities. Such strategies warrant greater focus.

## Data availability statement

The raw data supporting the conclusions of this article will be made available by the authors, without undue reservation.

## Ethics statement

The studies involving humans were approved by the Ethics Committee of Shanghai University of Traditional Chinese Medicine Affiliated Yueyang Hospital of Integrated Traditional Chinese and Western Medicine. The studies were conducted in accordance with the local legislation and institutional requirements. The participants provided their written informed consent to participate in this study. Written informed consent was obtained from the individual(s) for the publication of any potentially identifiable images or data included in this article.

## Author contributions

WL: Conceptualization, Methodology, Project administration, Writing – original draft, Writing – review & editing. GZhu: Data curation, Formal analysis, Visualization, Writing – review & editing. YL: Data curation, Investigation, Project administration, Writing – review & editing. JW: Investigation, Project administration, Writing – review & editing. ZF: Investigation, Project administration, Validation, Writing – review & editing. JT: Data curation, Formal analysis, Investigation, Project administration, Writing – review & editing. GZha: Conceptualization, Resources, Validation, Writing – review & editing. DX: Conceptualization, Supervision, Writing – review & editing.
